# Tending to the Facial Surfaces of a Mathematical Biology Head-Scratcher: Why Does the Head of the Sea Turtle *Natator depressus* Resemble a Convex Zygomorphic Dodecahedron?

**DOI:** 10.3390/ani15010100

**Published:** 2025-01-04

**Authors:** David A. Becker

**Affiliations:** Department of Chemistry and Biochemistry, Florida International University, Miami, FL 33199, USA; beckerd@fiu.edu

**Keywords:** anatomy, head, segmentation, geometry, topobiology

## Abstract

Two convex polyhedra that markedly resemble the head of the flatback sea turtle hatchling are identified. The first example is a zygomorphic tetragonal dodecahedron, while the other, an even better matching structure, is a related tetradecahedron, herein speculated to arise from this particular dodecahedron via known mechanisms gleaned from studies of the behavior of foams. A segmented, biomorphic, convex polyhedral model to address cephalic topology is thus presented stemming from solid geometry, anatomical observations, and a recently computed densest local packing arrangement of fifteen slightly oblate spheroids in which fourteen oblate spheroids surround a central such spheroid. This particular array of oblate spheroids shares salient structural features with the aforementioned dodecahedron. Successful testing of the model has been achieved by converting this array of fifteen oblate spheroids constructed with putty to the cephaloid dodecahedron in a process involving ventral elongation induced by stretching in the anterior direction along the anteroposterior axis (convergent extension). During convergent extension, the two left most anterolateral oblate spheroids that are in direct contact with the ventral spheroid of the array merge into a single lateral facet of the incipient dodecahedron, while the corresponding two right such oblate spheroids do the same. Thus, the fourteen outer oblate spheroids of the array give rise to the twelve facets of the finalized dodecahedron, while the central oblate spheroid remnant assumes an interior dodecahedral position. The hypothetical dodecahedron to tetradecahedron transformation entails the collapse of a tetravalent vertex (which is known to occur in foams as part of a T1 transition) followed by bilateral facet splitting. Remarkably, a model stipulating that convexity is to be retained in connection with this sequence of steps necessitates that the starting dodecahedral template undergoes modification to become a tetradecahedron in possession of precisely the highly ordered feature found at the top of the head of numerous specimens of the flatback sea turtle hatchling, namely, a fused medial pentagon–heptagon pair in the form of a pentagonal frontal scute and heptagonal frontoparietal scute. Such a possible new instance of geometric biomorphy, taken together with the correct anticipation of the cephalic pentagon–heptagon pair, might serve to instill further confidence in renewed efforts to shed light on morphogenesis with foam embryo models.

## Simple Summary

The shape and exterior segmentation of the head of the flatback sea turtle hatchling substantively matches the topology of an unusual zygomorphic polyhedron comprised of twelve planar quadrilateral facets and, more so, a related tetradecahedron variant featuring the striking dorsal pentagon–heptagon pair which constitutes the edge-sharing pentagonal frontal scute and heptagonal frontoparietal scute that is seen in numerous specimens of this animal. Remarkably, though speculatively, close packing of fifteen identical slightly flattened spheres constructed with an elastic putty can generate this dodecahedral pattern upon stretching along the central axis. This dodecahedral motif is hypothesized to give rise to the aforementioned tetradecahedral patterning via known processes of foam dynamics. Thus, in what may signify a potentially novel instance of geometric biomorphy, the highly ordered bilaterally symmetrical convex tetradecahedron of ten quadrilateral facets, three pentagonal facets, one heptagonal facet, nineteen vertices, and thirty-one edges described herein is visibly cephaloid, and its proposed formation might serve as yet another line of evidence to further bolster the resurgent attention on foam embryo models that is a focus of a cohort of contemporary developmental biology research groups.

## 1. Introduction

The process by which animals and other multicellular organisms assemble from a single spherical cell to achieve three-dimensionally complex bauplans has intrigued and challenged investigators for centuries. It is one of the most confounding lines of inquiry in all of science that has, after diligent research, brought to light astounding phenomena such as the embryonic induction associated with classic experiments in newts with the Spemann–Mangold organizer [1]. As Dalcq fervently pointed out in 1938, the escape from sphericity during embryogenesis is a fascinating struggle [2]. Such a feat requires symmetry breaking in order to disrupt the vaunted symmetric perfection inherent to the sphere [3]. The role of mechanics in morphogenesis, while somewhat falling out of favor due to impressive advances in the understanding of genomic information toward the middle and latter part of the twentieth century (such as the early morphogen and homeobox-containing protein stemming from the bicoid gene of the fruit-fly *Drosophila melanogaster* [4]), has recently regained significant attention to reinvigorate that to which, in 1888, Wilhelm Roux assigned the German term Entwicklungsmechanik, or developmental mechanics [5].

At various points since the inception of nineteenth century developmental mechanics, the recognition that the behavior of multicellular aggregates in tissue might be similar to that of soap bubbles in foams has served as a springboard to assist in the search for insights into morphogenesis. One of the earliest allusions to the potential bio-morphological significance of foams is that of D’Arcy Thompson’s discussion of soap bubbles in his 1917 treatise *On Growth and Form* [6]. Subsequently, other bio-scientists have pursued the possible role of foams in developmental biology, such as Richard Gordon [7], Denis Weaire [8], and Stuart Newman [9]. Very recently, independent reports from the research groups of Otger Campas [10], Lisa Manning [11], and Herve Turlier [12] have described their progress in formulating successful foam embryo models. Another notable recent publication in this regard is from Sharon Lubkin’s research group and documents the embryonic importance of bubble packing in the morphogenesis of the notochord [13].

The aforementioned 2021 manuscript from the Campas group is entitled *Embryonic tissues as active foams* [10]. Within this work, a featured process incorporated into the model is that of foam dynamics in elongating tissue. With respect to the mechanics of embryogenesis, elongation of tissue and the attendant cell rearrangements affiliated with convergent extension [14] have been extensively examined and reviewed by Lance Davidson and his research group in the course of their studies of embryo mechanics at the University of Pittsburgh [15]. A particular type of rearrangement within cell clusters during convergent extension that has been scrutinized by Davidson (and that will be featured subsequently within the present work) is a foam process known as a T1 transition or neighbor exchange, first described by Weaire in 1984 [16]. In 2023, Turlier and colleagues, as pointed out above [12], introduced their foam embryo model in the form of a software package entitled “foambryo” that is based upon maps of the configurations of cells in early embryos as a consequence of internal pressure and heterogeneous interface tensions of individual cells of the aggregate. The recent foam embryo model contributions by both Campas and Turlier contain discussions of T1 transitions.

Geometric objects can be reminiscent of biological structures (geometric biomorphy). Such an instance of geometric biomorphy in D’Arcy Thompson’s *On Growth and Form* [6] involves the recounting of the verbal reaction of the operator of an industrial crane upon seeing an anatomical rendering of a human femur with its aligned trabeculae. Upon inspection of the anatomical drawing, the operator exclaimed something to the effect of “That’s my crane!”. That the crane and femur were similar was taken as evidence in support of the notion that biological morphology settles upon a form that is optimized to withstand load forces, and that this optimized form is mirrored within the geometry and design of the anthropogenic load-bearing machine. In 1931, a publication in Nature by Thompson appeared with the title *Embryology and the Theory of Polyhedra* [17].

Another important morphogenesis theory that has gained some traction with respect to efforts to account for the geometric patterns observed in the carapace of turtles is Turing’s reaction–diffusion model [18] as summarized in a recent review article [19]. However, as of yet, there appears to be no evidence of successful application of this model to cephalic patterning in testudines or other vertebrates despite the identification of signals contributing to the formation of scutes and, more generally, the epidermis in sauropsids [20,21,22]. The issue of the origin of head morphology in vertebrates remains unsettled despite many intensive inquiries on head segmentation, such as those of the research group of Shigeru Kuratani [23]. The Turing mechanism, while previously assumed to be operative in establishing the regularly repeating geometric patterning of ommatidia, has since been supplanted with new biomechanical evidence [24]. Recently, a reaction–diffusion model has been employed to rationalize the hexagonal patterning of the scales on the non-cephalic portion of the bodies of snakes [25].

In terms of factors contributing to biological morphology, the minimization of surface area via close packing of cells has received considerable attention [26]. Studies noting the hexagonal packing in liver lobules [27] and ommatidia [28] are two such well known examples. Recently, Sato and coworkers have discovered that a transition of ommatidia from hexagonal packing to tetragonal packing can be induced by stretching [29]. Furthermore, while the compound eye of certain animals displays hexagonal packing, tetragonal packing is seen in others, such as in certain crustaceans. The fact that stretching can result in a shift from hexagonal packing to a tetragonal arrangement of ommatidia may lend credence to a speculative model that is proposed to account for the instance of geometric biomorphy that is highlighted within the present research. Additionally, Lubkin’s recent and aforementioned connection of bubble packing to the morphology of the notochord is another interesting geometric biomorphy example [13].

**Figure 1:**
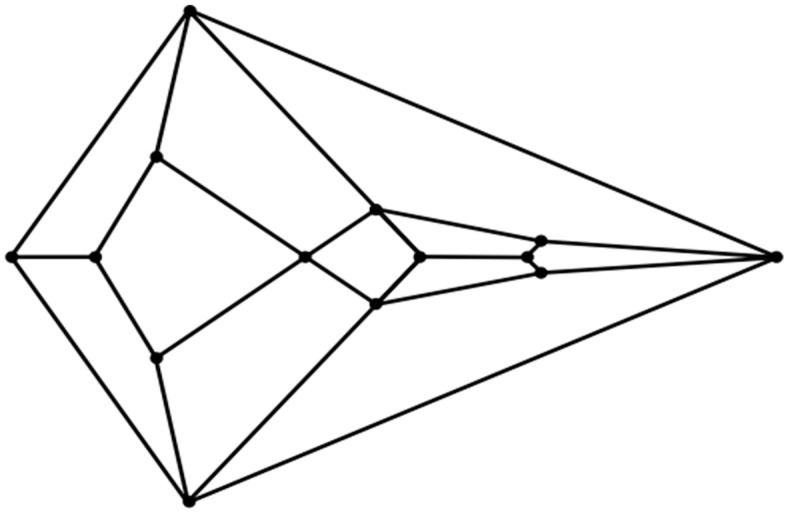
Plane projection of the convex cephaloid tetragonal dodecahedron.

**Figure 2:**
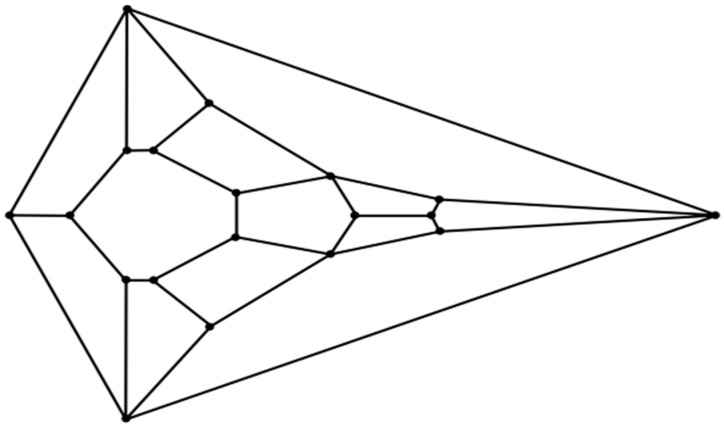
Plane projection of the convex cephaloid tetradecahedron.

**Figure 3:**
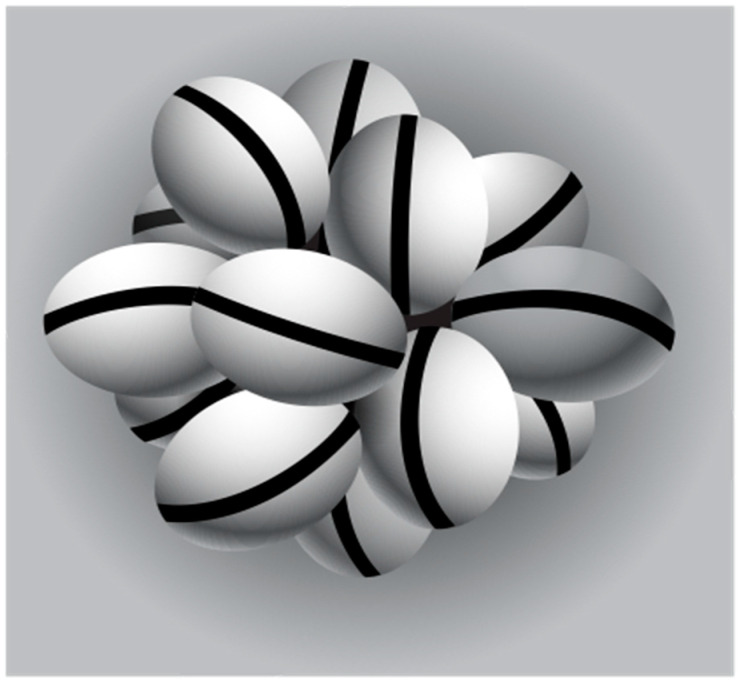
Densest local packing array of fifteen slightly oblate spheroids.

Can the vertebrate head be compared to a polyhedron? In 1969, Jardine reported his efforts to find homology between the complex head segmentation patterns of different species of fossilized fish [30]. Within this article, anatomical drawings in which the heads of these fish are projected upon a plane are carefully rendered. The number of non-convex polygonal segments in these drawings ranges from about twenty to forty. With respect to the perimeters in these meticulous drawings, they are non-convex. As Jardine’s intent was not to compare the segmentation patterns in these fish heads to that of a convex polyhedron, he had no reason to impose convexity. Indeed, any attempt to do so in fish or other animals must account for the existence of some anatomical structure (such as a neck) to provide a surface connecting any such convex cephaloid polyhedron to the rest of the body. But what if the identification of an animal head that strongly resembles the features of a convex polyhedron is actualized? Would such a discovery of geometric biomorphy be simply a coincidence, or perhaps something more? Given the high degree of complexity that is commensurate with cephalogenesis, skepticism in such a case would certainly be judiciously warranted. Nevertheless, speculation has its place in scientific endeavors regardless of the daunting difficulties which accompany attempts to understand an exceedingly puzzling process. This paper will document an instance in which the head of the hatchling of an extant sea turtle species, namely, the flatback sea turtle (*Natator depressus*), does indeed resemble the features of an unusual convex zygomorphic dodecahedron (projected onto a plane in the diagram in Figure 1) and, all the more, the features of a conceivably ensuing tetradecahedron variant (projected onto a plane in the diagram in Figure 2). Despite its speculative nature, an attempt to explain the morphogenesis of such cephaloid polyhedra will be made as a potential ramification of a recently computed densest local packing arrangement of fifteen slightly oblate spheroids that is nearly spherical (Figure 3), and a treatment of such spheroids as an array of deformable viscoelastic entities that undergo shape changes upon convergent extension coupled with established transitions from foam dynamics. Thus, a key aspect of the argument proposed herein is that the featured cephaloid dodecahedron and cephaloid tetradecahedron may be seen to arise in rational steps from a spherically or quasi-spherically symmetrical origin.

## 2. Materials and Methods

The putty used to model the formation of the dodecahedral pattern in Figure 1 is the commercially available silicone-based children’s putty marketed as “Silly Putty”. The polydimethylsiloxane (PDMS) in this commercial putty is, among other investigated surrogates, known to serve as a particularly suitable simulant for soft tissue [31]. Fifteen putty oblate spheroids of approximately 3.0 cm in diameter and 2.7 cm in height were prepared and placed by hand so as to approximate the array in Figure 3. Stretching and anterior pinching to mimic convergent extension were conducted manually.

## 3. Results

**Figure 4:**
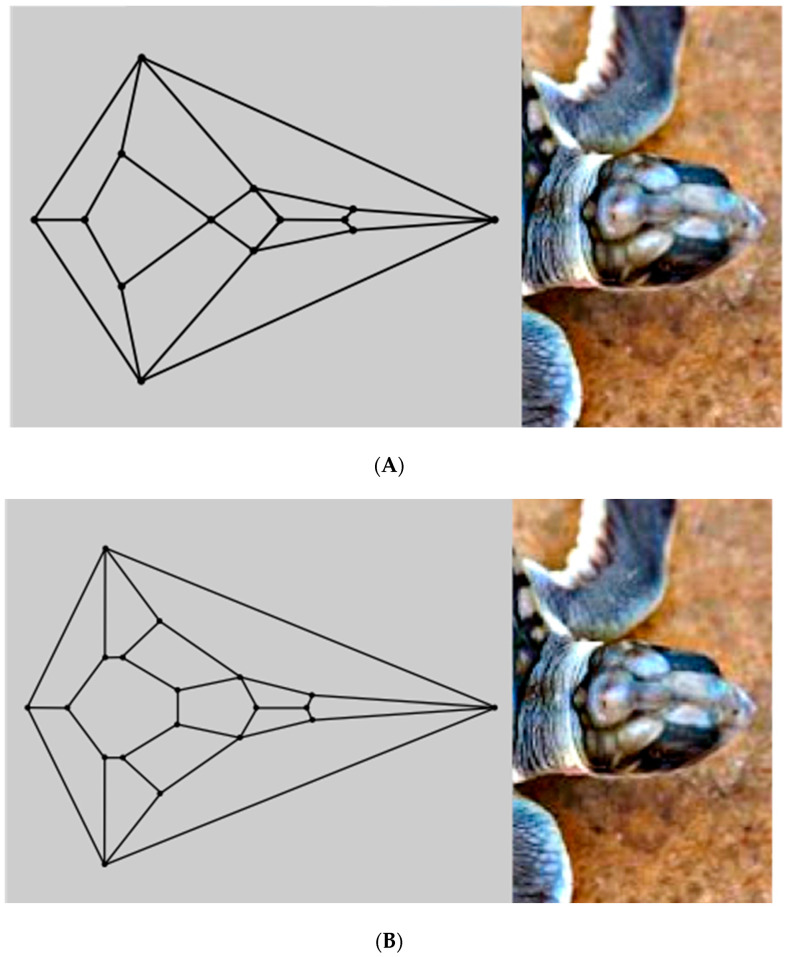

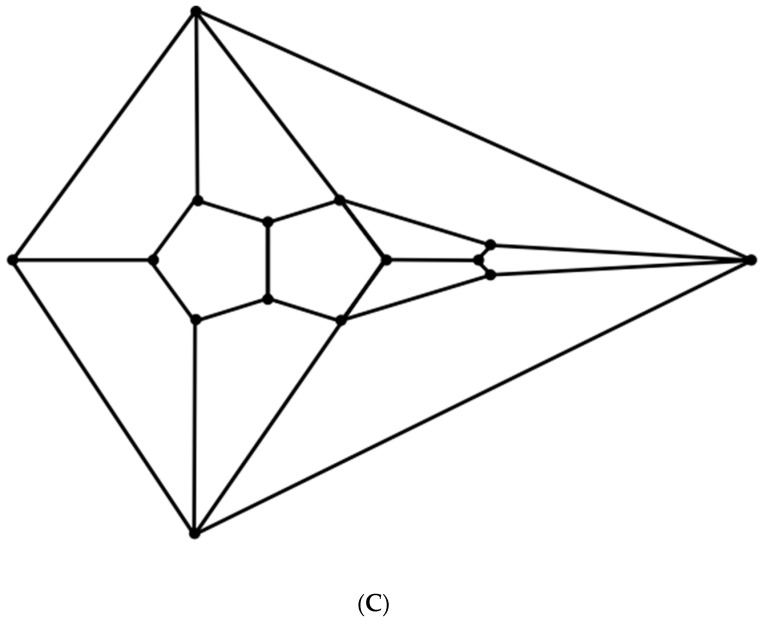
(**A**) Correspondence between the plane projection of the cephaloid dodecahedron and the patterning in the head of the flatback sea turtle hatchling. (**B**) Correspondence between the plane projection of the cephaloid tetradecahedron and the patterning in the head of the flatback sea turtle hatchling. (**C**) A dodecahedral relative of the featured tetragonal dodecahedron arises with a fused pentagon–pentagon pair after a transition known to occur in foams is performed.

An inspection of the head of the flatback sea turtle hatchling notably reveals a striking similarity to the unusual convex zygomorphic dodecahedron that is comprised of twelve quadrilateral facets, as can be gleaned from Figure 4A, in which the dodecahedron is represented by projecting it onto a plane through its large bottom facet, such that these four basal edges constitute the perimeter. This dodecahedron’s connectivity is neither that of any Catalan solid, Johnson solid dual, nor trapezohedron and, remarkably, to the best of the author’s knowledge, is a yet unnamed array of quadrilaterals, unlike the connectivity seen in the more familiar rhombic dodecahedron or trapezo-rhombic dodecahedron.

An even better topological match between the head of the flatback sea turtle hatchling and a convex polyhedron than that involving the aforementioned dodecahedron is that involving the related Figure 2 tetradecahedron, as seen in Figure 4B. A possible process to convert the dodecahedron in Figure 1 to the tetradecahedron in Figure 2 starts with the development of a new edge between the two quadrilateral facets corresponding to the frontal scute and more caudal frontoparietal scute of the flatback sea turtle hatchling head as the frontoparietal spheroid matter encroaches upon the jammed frontal spheroid matter during convergent extension. Such a process would convert each of these two quadrilateral facets into pentagonal facets of the dodecahedron represented by the diagram in Figure 4C and has precedent in foam dynamics as part of a T1 transition. Overall, the new edge formation increases the number of vertices in the polyhedral variant by one. As the edge develops during this rearrangement, the two other quadrilateral facets that initially shared the tetravalent vertex participating in the transition are pushed laterally away from one another. Bilateral facet splitting with the creation of two new facets, four new vertices, and six new edges converts the dodecahedron in Figure 4C to the tetradecahedron in Figure 2. Such facet splitting can perhaps be rationalized as a ramification of forces acting in the supraocular region as the subjacent ophthalmic tissues of the large bulging eye induce distortions from planarity on each of the bilateral facets of the Figure 4C dodecahedron best corresponding to the supraocular region, with the production of two new quadrilateral facets in a bilateral manner. If the imposition of convexity is maintained in this model approximation, then the creation of these two new quadrilateral facets transforms the pentagonal facet corresponding to the frontoparietal scute in Figure 4C to a heptagon. Each instance of this type of facet splitting in a convex tetragonal polyhedron generates one additional facet, two additional vertices, and three additional edges in a convex polyhedral offspring. For example, the facet splitting of one of the six square facets of a cube into two congruent rectangular facets necessarily generates an irregular pentagonal prism if it is stipulated that convexity is retained in the ensuing polyhedron with seven facets. In foam dynamics, bubble splitting can indeed occur under the auspices of mechanical forces, such as shear [32]. A recent review on the morphology of supraocular scales in reptiles documents that the number of such scales can generally vary from only one to as many as eight over each eye. With respect to such supraocular scale cracking, the invocation of a cracking mechanism in head scales may bear some parallels to what is known to operate in terms of shear-like forces during the establishment of the non-serial cracking patterns that are observed in crocodile heads [33,34].

**Figure 5:**
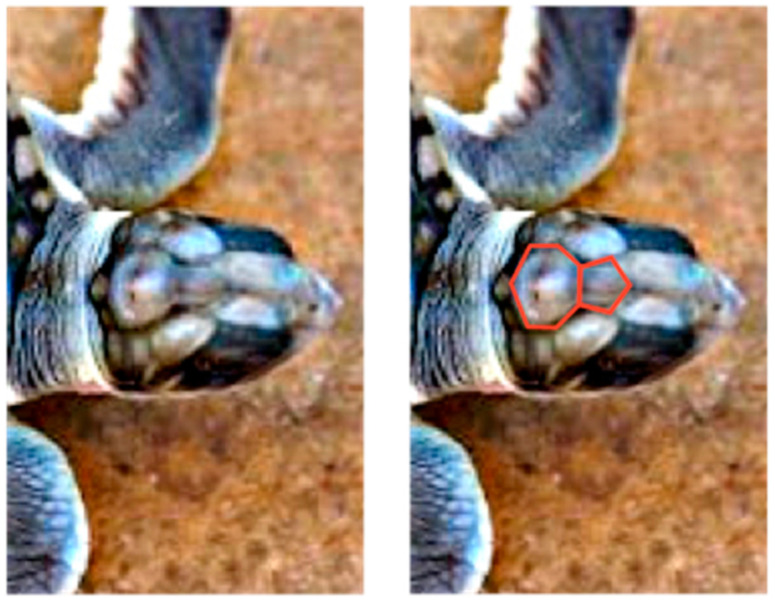
Highlighting (in red) of the pentagon–heptagon pair on the flatback sea turtle hatchling head.

**Figure 6:**
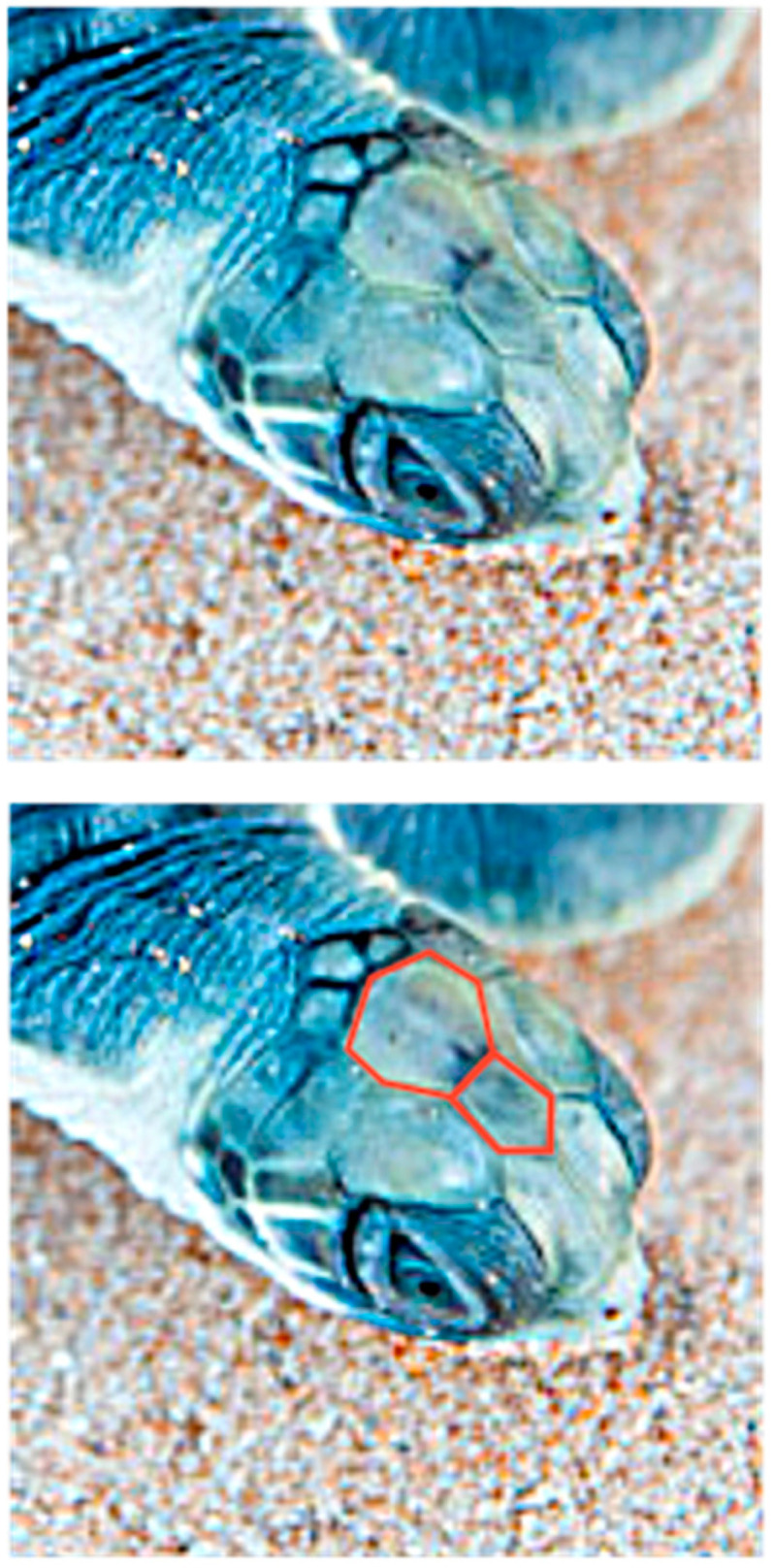
A clearly heptagonal frontoparietal scute morphotype in a flatback sea turtle hatchling specimen (fused to a pentagonal frontal scute).

**Figure 7:**
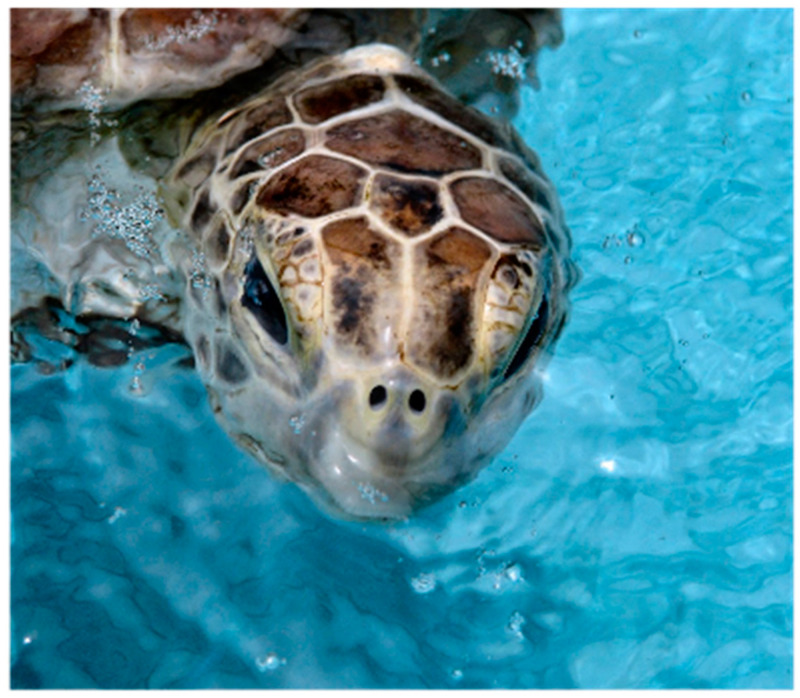
A specimen of the green sea turtle (*Chelonia mydas*) exhibiting a clearly heptagonal frontoparietal scute fused to a pentagonal frontal scute.

The unusual cephaloid tetradecahedron in Figure 2 possesses 64 discrete elements (14 facets, 19 vertices, and 31 edges). Of the fourteen facets, ten are quadrilaterals, three are pentagons, and one is a heptagon. Remarkably, the model contemplated herein, commencing from the dodecahedral template in Figure 1, accurately predicts the biomorphic presence of the rather ornate fused medial pentagon–heptagon pair morphotype (in which a pentagonal facet corresponding to the frontal scute is fused to a heptagonal facet corresponding to the frontoparietal scute) in the Figure 2 tetradecahedron. The frontal scute of the flatback sea turtle hatchling as seen in Figure 4A is easily construed as pentagonal and the frontoparietal scute as fairly heptagonal, as highlighted in Figure 5. While there is some intra-species variation in the morphology of the frontoparietal scute of flatback sea turtle hatchlings, perusal of multiple images of flatback sea turtle hatchling heads in the public domain reveals numerous specimens with the fused medial pentagon–heptagon pair morphotype, in which a clearly heptagonal frontoparietal scute is seen, as can be observed in the specimen shown in Figure 6. Likewise, the curated image found on the Smithsonian Institution’s National Museum of Natural History website for the flatback sea turtle [35] displays specimens, such as the hatchling specimen in the lower left corner of this image, with a readily discernible convex heptagonal perimeter demarcating the frontoparietal scute. Moreover, the green sea turtle (*Chelonia mydas*), the only other of the world’s seven extant sea turtle species to possess, like the flatback sea turtle, a single pair of prefrontal scutes, can be seen in Figure 7 to overtly exhibit the flatback sea turtle’s same rostral to caudal fused medial pentagon–heptagon pair morphotype in terms of its frontal scute (pentagonal) and frontoparietal scute (heptagonal).

Having addressed the keen resemblance between the head of the flatback sea turtle hatchling and the tetradecahedron represented in Figure 2, as well as a hypothetical pathway from the dodecahedron in Figure 1 to the Figure 2 tetradecahedron, the results of a model study, in which the generation of the Figure 1 dodecahedral connectivity occurs from the computed densest local packing array of fifteen slightly oblate spheroids, are worthy of consideration. Thus, as alluded to above, a report in the recent physics literature by Kapfer et al. [36] contains computational studies with respect to the determination of the kissing number and densest local cluster structures of congruent uniaxial ellipsoids as a function of the aspect ratio. In regard to biological morphology, a particularly noteworthy structure discovered by Kapfer et al. may be that in which fourteen slightly oblate spheroids (with aspect ratio of approximately 0.9) surround a fifteenth such spheroid as shown in Figure 3. The Figure 3 structure, with its 1-6-6-1 outer layering, is reminiscent of the positions of the facet centers of many tetradecahedra, such as the truncated hexagonal trapezohedron (a curved version of which is one of the two polyhedral components of the famous Weaire–Phelan foam packing paradigm [37]), and also bears considerable similarity to the dodecahedron represented in Figure 1.

In order to model a putative pathway to the Figure 1 cephaloid dodecahedron from the assemblage of fifteen oblate spheroids in Figure 3, it may prove useful, as has been pointed out in recent biological studies, to imagine the oblate spheroids as soft viscoelastic matter in the form of a putty [38]. These putty spheroids can undergo coalescence with neighbors in analogy to what is well documented for tissue fusion during morphogenesis and 3D bioprinting with tissue spheroids [39]. The capacity of such multicellular spheroids to merge with living tissue in humans is known, as has been recently demonstrated with the therapeutic cartilage repair treatment Spherox [40]. Many such multicellular spheroids have been shown to possess a slight oblateness [41] that would be needed to arrive at the Figure 3 structure, and investigations of organoids document aggregates of cell clusters that feature topologies replete with budding [42], as would also be required in the contemplated model.

The 1-6-6-1 arrangement of the four layers of the fourteen outer spheroids of the Figure 3 array is such that these fourteen spheroids have positions that can be theorized to correspond to cephalic embryological structures of vertebrates (such as those of a chick embryo at stage HH15). Thus, the top spheroid in the first outer layer of the model can be thought to correspond to the frontonasal prominence at the forehead region (the segment including the anterior forebrain that is formed upon final closure of the anterior neuropore). In the second outer layer, the most anterior of the six spheroids has a dorso-rostral snout position that best matches the medial nasal process (a precursor of the intermaxillary segment), while its two flanking spheroids in the second layer are assigned to the paired lateral nasal processes. The posterior spheroid in the second layer is ascribed to the mesencephalic vesicle, with the remaining two spheroids in the second layer comprising forebrain components with paired optic cups. Of the six spheroids in the third layer, the two posterior spheroids represent the two sides of the metencephalic vesicle of the developing hindbrain, the two most rostral spheroids in this layer correspond to a bilateral premandibular region in the vicinity of the apex of the primitive maxilla, and the two remaining third outer layer spheroids correspond to bilateral precursors of the maxillomandibular junction. The single bottom spheroid in the fourth outer layer represents tissue from the first pharyngeal arch that will ultimately become the mandibular process (with later bilateral contribution of some tissue to the superjacent third outer layer maxillomandibular junction spheroids in what will arise as the paired maxillary processes). The central oblate spheroid best corresponds to primordia of the medial basicranium.

**Figure 8:**
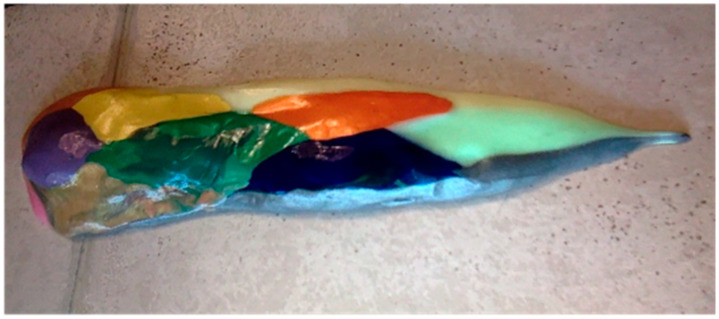
The connectivity of the cephaloid dodecahedron is seen in the dorsal view of the model obtained from the Figure 3 array of fifteen putty oblate spheroids after stretching has been conducted to mimic convergent extension.

Upon constructing an array of fifteen putty oblate spheroids according to the favored juxtapositions within Figure 3, it is possible, as shown in Figure 8 (dorsal view), to achieve a transformation to the eleven facets comprising the complete hull of the biomorphic dodecahedron in the Figure 1 representation by a mechanism involving stretching of the front of the hexagonal layer which articulates the most ventral spheroid in the anterior direction along the axis that is contained in the lone reflection plane of the array (the anteroposterior axis). This stretching is intended to mimic convergent extension [43], a process which also accounts for compressive forces that give rise to anterior narrowing. Morphogens that promote ventral elongation have been described in studies of craniofacial development [44].

**Figure 9:**
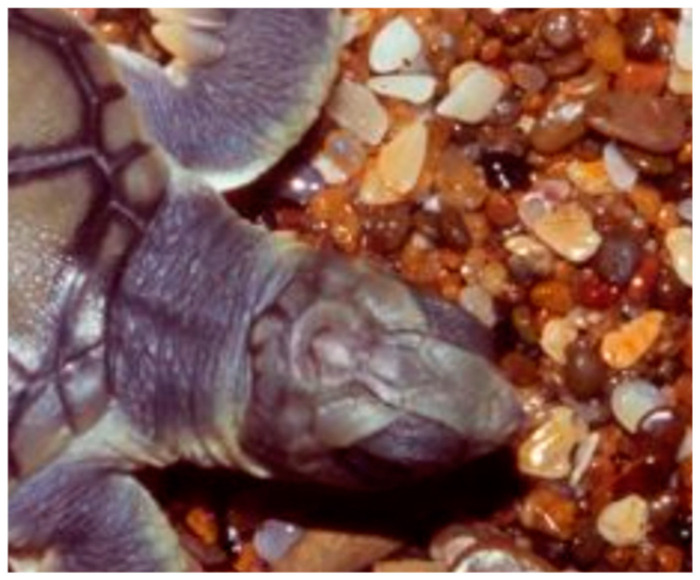
A prominent fused medial pentagon–heptagon pair is seen atop the head of this flatback sea turtle hatchling specimen, as well as the overall tetradecahedral cephalic patterning that arises from the dodecahedral connectivity of the putty model.

As the conversion of the densely packed oblate putty spheroids in the model to the hull of the cephaloid dodecahedron proceeds, the ventral elongation, in a possible analogy to the embryological significance of the movements of the maxillary processes, causes two of the left most anterolateral spheroids in the lower hexagonal outer layer (two white putty oblate spheroids were used as such) to eventually merge into a single lateral facet (the white facet in Figure 8) that ultimately shares the most anterior midline vertex of the resulting assembly, and the corresponding two right anterolateral spheroids (two gray putty oblate spheroids were used as such) do the same (producing the gray facet in Figure 8) to thereby establish the two anterolateral edges of the large quadrilateral kite-shaped base of the polyhedron. Animals, such as certain turtles, snakes, and geckos, are indeed adorned with kite-shaped or diamond-shaped heads with a clear quadrilateral perimeter. In what may be analogous to the embryological migration and midline convergence of the nasal placodes, the anterior narrowing also brings the two most anterior spheroids of the upper hexagonal outer layer of the array into contact. Such contact results in the formation of an edge that is shared by the two facets of the resulting polyhedron that correspond to the two prefrontal scutes of the flatback sea turtle head (the dark blue and orange facets in Figure 8). It is also conceivable that these two particular oblate spheroids are brought into contact by their proliferation, or by a combination of proliferation and anterior narrowing. The stretching forces of convergent extension also serve to expand the lone putty spheroid at the bottom of the array into the basal facet of the cephaloid dodecahedron corresponding to the mandible, a structure which is derived from tissue that emanates from the first pharyngeal arch. The high resolution image in Figure 9 shows the apparent tetradecahedral head of yet another flatback sea turtle hatchling specimen, and its overtly discernible pentagon–heptagon pair arising hypothetically from the dodecahedral connectivity in the putty model.

## 4. Discussion

The role of stretching in morphogenesis was long ago highlighted in the transformation grids of D’Arcy Thompson [6]. An iconic example, albeit controversial [45,46], illustrates the use of such grids to transform a porcupinefish (*Diodon hystrix*) into a sunfish (*Mola mola*). Subsequently, the technique of Bookstein morphing [47] has also generated widespread attention as an important tool for the assessment of homology when comparing the disparate morphologies of related species.

A crucial embryological event that is driven by stretching is that of convergent extension [14,43]. Much work has been expended toward deciphering the myriad mechanical and/or chemical factors which play a role in convergent extension as it brings about tissue elongation. Expounding upon the issue of the imposition of a convex quadrilateral perimeter in modeling the head of the flatback sea turtle hatchling head, such a kite-like perimeter can be seen as a logical ramification of the effects of convergent extension on a closest packed 2D array with standard six-around-one hexagonal packing [48]. Thus, imagine a set of six thin identical strips of cork that are attached to six inverted thumbtacks upon a table with the vertex positions of a regular hexagon, so that the six cork strips now form the six edges of the regular hexagon. Upon immobilizing any two adjacent edges (which shall be attributed to the two most caudal cephalic edges), a stretching force to mimic convergent extension that is applied to the most rostral vertex in the anterior direction along the anteroposterior axis will transform the polygonal perimeter from that of a regular hexagon to that of a kite-shaped quadrilateral in which both of the anterior edges are twice the length of either of the two posterior immobilized caudal edges. In this construct, the rationale for immobilization of the two most caudal edges is that these two edges will be most resistant to change positions because they correspond to caudal cephalic regions that are anchored to the articulating non-cephalic posterior portion of the embryo.

It is interesting to note that, with the admittedly speculative assumption that the dodecahedral to tetradecahedral lineage is valid, there is another possible mechanism for splitting the putative initially unified supraocular scutes other than buckling due to the presence of the large bulging eyes underneath these head shields. Thus, the stretching which is affiliated with convergent extension could potentially lead to the splitting of the supraocular scutes, in view of recent evidence documenting that mechanical strain on an embryo can cause elongation followed by splitting of tissue units (somites) with the formation of a nascent boundary between these two newly formed units of the division [49].

Whether such conjectures are true or false does not change the fact that a pathway that starts with the convex cephaloid dodecahedron represented by the 2D projection in Figure 1 and then proceeds via the aforementioned tetravalent junction collapse which accompanies a T1 transition in foams, followed by bilateral facet splitting to produce the two additional facets, or vice versa, will result in a tetradecahedron that, if convex, is represented by the 2D projection in Figure 2—a projection that is conspicuously akin to the connectivity exhibited in the head of the flatback sea turtle hatchling—and which especially contains the intriguing feature of a starkly matching rostral to caudal fused medial pentagon–heptagon pair. That this unusual cephalic structure is precisely what is observed in specimens of both the flatback sea turtle and the green sea turtle is perhaps particularly notable. To the best of the author’s knowledge, no other hypotheses of vertebrate cephalogenesis have yet been proffered (such as Johann Wolfgang von Goethe’s early vertebral theory of the skull [50]) that would yield the expectation of this elaborate azulenoid pentagon–heptagon tiling.

The possibility of generating two plates from an initially unitary supraocular plate via facet splitting is not without precedent in reptiles. Thus, a recent report on the aquatic snake *Cerberus schneiderii* delineates intraspecies variability in the plate morphology of the supraocular region [51]. Some specimens of this snake have a single supraocular plate, while other specimens display a fragmented supraocular morphotype with two supraocular plates above each eye, as can be seen in Figure 7A,B of that manuscript. Moreover, the specimen with the fragmented supraocular morphotype in Figure 7B appears to have a larger eye than the specimen in Figure 7A. The so-called “supraocular bulge” of many reptiles and amphibians [52] is a structural element that, in some such animals, substantially deviates from convexity in that region and the subjacent ocular tissues of a large eye may be thought to exert forces to precipitate such facet splitting with the appearance of one, or sometimes several, new sutures within the field of the initially larger plate. Another such example was noted by the Dutch herpetologist Leo Brongersma in 1947 wherein he observed that the Sumatran blood python (*Python curtus Schlegel*) has variability of supraocular plates such that a certain specimen “has three supraoculars on the right side, of which the middle one is very narrow: on the left side this specimen has only one supraocular, which has two short incisures in its medial border, thus also pointing to a division into three shields” [53].

If, as speculated, the tetragonal dodecahedron represented by the Figure 1 diagram were to serve, in actuality, as a predecessor to the Figure 2 tetra-decahedron, then, evolutionarily, it would not be unreasonable to expect that the dorsomedial pair of quadrilaterals that share one tetravalent vertex in the Figure 1 diagram could be found as a reptilian morphotype among the plethora of such animals. Interestingly, the blue-throated rainbow skink (*Carlia rhomboidalis*), a lizard indigenous to Australia, does indeed possess, as seen in the images of this animal on the repfocus.dk website (https://repfocus.dk/natureswindow/Reptilia/Carlia_rhomboidalis.html accessed on 8 October 2024), this particular structural feature from which, etymologically, the animal’s rhomboid frontoparietal scale is presumed to have led to its designated species name [54]. Furthermore, if, as speculated, the aforementioned tetravalent vertex were, in actuality, to collapse with the formation of a new edge via the second stage of a T1 transition, then a morphotype in which two pentagons in the form of two dorsomedial plates that share an edge that is orthogonal to the anteroposterior axis (a configuration contained in the Figure 4C dodecahedron) would be evolutionarily implicated as a likely anatomical cephalic structural feature. It is worth noting that specimens of the common wall lizard (*Podarcis muralis*) found in Poland display a pattern that is strongly suggestive of a pentagon–pentagon fusion in precisely this way, as seen in Figure 5 of that manuscript [55]. Thus, the perimeter of the frontal scale of three such specimens in this image is that of a non-convex octagon that matches the morphology of the pentagon–pentagon fusion which exists in the perimeter defined by the eight carbon atoms of the organic molecule pentalene. A fourth specimen in this image (the specimen in Figure 5c) does indeed present a central edge which separates the frontal scale into two medial and nearly identical fused pentagonal regions (there is a small additional asymmetric edge in the posterior pentagonal region). With regard to the fact that *Podarcis muralis* typically occurs with two supraocular plates above each eye yet lacks the heptagonal frontoparietal plate seen in both the flatback and green sea turtles, one possible explanation is that the supraocular bulge of this lizard is of such large magnitude that it brings about a deviation from convexity so pronounced that a split of the supraocular field into two scales does not lead to the pentagon to heptagon conversion which is expected with adherence to a convex (or nearly convex) topology. Likewise, even though the supraocular region of *Carlia rhomboidalis* is typically more highly fragmented into four supraocular plates above each eye, the high degree of non-convexity due to copious supraocular bulging in this animal might also prevent the rhomboid frontoparietal plate from gaining additional edges during supraocular fragmentations.

## 5. Conclusions

The complexities of vertebrate cephalogenesis are profoundly difficult to unravel. Nevertheless, it is surprising and zoologically interesting to find a possible new example of geometric biomorphy in a convex polyhedron that so keenly matches the topological features of the cephalic portion of an extant reptile. The cephaloid tetradecahedron described herein has 63 of its 64 elements on display in its dorsal 2D projection (it is only the ventral facet corresponding to the mandibular region that is occluded from the viewer in such a perspective). Careful inspection of the head of the flatback sea turtle hatchling, as seen in the image in Figure 4B, allows for the identification, with proper connectivity, of 62 of the 63 elements expected to be visible (13 of the 14 facets are visible as expected, all 19 vertices are visible, and 30 of the 31 tetradecahedral edges are visible). Thus, the identification success rating is greater than 98% (62/63 = 0.984). The lone tetradecahedral edge that is not readily apparent is that which connects the most caudal of the 19 vertices to what corresponds to the most caudal vertex of the heptagonal frontoparietal scute. It is worth noting that such an edge is plainly visible in the head of the closely related sea turtle *Chelonia mydas* (the green sea turtle). The unanticipated ability of the convex dodecahedral template in Figure 1 to seemingly account for, in a conceivably ensuing tetradecahedron, the unusual dorsally situated rostral to caudal fused medial pentagon–heptagon pair of the discernibly tetradecahedral flatback sea turtle hatchling head (a feature also at the top of the head of *Chelonia mydas*) is a key highlight of this work that is, to the best of the author’s knowledge, without any attempts at an explanation elsewhere. The existence of this pentagon–heptagon pair and its possible genesis as described via established pathways of foam dynamics could potentially be seen to render prescient D’Arcy Thompson’s discussions of soap bubbles in *On Growth and Form* and may constitute yet another phenomenon to lend impetus in support of the ongoing efforts of current investigators to pursue foam embryo models.

Is there any structural advantage to a convex or nearly convex bio-architecture? One such possible benefit may be that of polyhedral rigidity in connection with Cauchy’s Rigidity Theorem of 1813 [56]. The constituent units surrounding a braincase should not be prone to excessive jostling in order to preclude damage to the skull and its contents. An animal head with the intrinsic rigidity of a convex polyhedron would also be expected to provide increased odds of survival upon the attack of a predator. Indeed, reptilian osteoderms have been postulated to have a role in defense against predation and, interestingly, a recent article describes a model in which such lizard head shields affect cephalic mechanics by reducing overall strain on the skull during biting [57]. For bilaterians, a fairly rigid cephalic base in the form of a kite via convergent extension-mediated elongation of a six-around-one closely clustered hexagonal packing unit would constitute a significant symmetry breaking event in that the six coplanar C2 axes of the regular hexagon are reduced to the single C2 axis of the resulting kite [58]. This reduction in symmetry can perhaps be construed as assisting in Dalcq’s “escape from sphericity”.

A prominent contemporary voice in promulgating the earlier views regarding the possibility that studies of modules of tightly clustered identical (or nearly identical) multicellular units may yield clues to the morphogenesis of organisms has been that of Stuart Newman. In a manuscript entitled *Inherency* that was published in book chapter form in 2021 [59], he writes, “Regarding development, inherency means that certain structural motifs (e.g., tissue layers, lumens, segments, appendages) can be readily generated by physical organizing forces acting on tissue masses, with minimal programming by the genome”. A recent and cogent article by Fred Bookstein [60] with the title *Reflections on a Biometrics of Organismal Form* includes portions of that Newman quotation as well as the following salient Newman quote from the same Newman manuscript: “In sciences other than biology, it is commonly recognized that a fixed range of forms is inherent to every type of matter and that variability, where it exists, is only expressed within that range”. Centuries earlier, in 1658, Sir Thomas Browne wrote in his book *The Garden of Cyrus*: “nature Geometrizeth, and observeth order in all things” [61]. The oblate spheroids model depicted herein, despite its speculative content, to which a prudent skepticism is justified in the face of the immense challenges associated with efforts to comprehend morphogenesis, subscribes to Newman’s and Browne’s precepts and, even be it only a matter of sheer coincidence, employs fundamental mechanisms from the foam dynamics toolkit to capture a reasonable likeness of the head of the flatback sea turtle hatchling in the form of an interestingly patterned convex tetradecahedron not previously recorded.

## Data Availability

The original contributions presented in the study are included in the article, further inquiries can be directed to the corresponding author.
